# Dr. Von Ammon

**Published:** 1861-09

**Authors:** 


The celebrated ophthalmologist, VON AMMON, Physician to the King of Saxony, recently died at Dresden, at an advanced age.

